# Initial predictors for short-term prognosis in anti-melanoma differentiation-associated protein-5 positive patients

**DOI:** 10.1186/s13023-021-01705-8

**Published:** 2021-01-30

**Authors:** Qihua Yang, Tianfang Li, Xin Zhang, Kunlong Lyu, Shujun Wu, Yan Chen, Shengyun Liu, Zujiang Yu

**Affiliations:** 1 Department of Rheumatology and Immunology, the First Affiliated Hospital of Zhengzhou University, Zhengzhou, 450000 China; 2 Department of Urology, the First Affiliated Hospital of Zhengzhou University, Zhengzhou, 450000 China; 3 Department of Respiratory, the First Affiliated Hospital of Zhengzhou University, Zhengzhou, 450000 China; 4 Department of Radiology, the First Affiliated Hospital of Zhengzhou University, Zhengzhou, 450000 China; 5 Department of Infection Diseases, the First Affiliated Hospital of Zhengzhou University, Zhengzhou, 450000 China

**Keywords:** Anti-melanoma differentiation-associated protein-5, Interstitial lung disease, Clinically amyopathic dermatomyositis, Dermatomyositis

## Abstract

**Background:**

Anti-melanoma differentiation-associated protein-5 (anti-MDA5) positive patients are characterized by the high mortality rate caused by interstitial lung disease (ILD). We conducted a retrospective study to summarize the clinical features and identify the initial predictors for death in anti-MDA5 positive patients.

**Methods:**

We designed a retrospective cohort of anti-MDA5 positive patients. The demographic and clinical data recorded on first admission, as well as the outcomes during the first six months follow-up, were collected. Predictors of rapidly progressive ILD (RPILD) and poor outcomes were calculated using logistic regression models and Cox proportional hazard regression models, respectively.

**Results:**

A total of 90 anti-MDA5 positive patients were included in this study. Eighty-one (90%) patients presented ILD on admission and 35 (38.9%) patients developed RPILD subsequently. During the first six months of follow-up, 22 (24.4%) patients died of respiratory failure at an average time of 6.6 ± 5.9 weeks. Factors including disease duration < 2 months (OR 6.1, 95% CI 1.7–22.4, *P* = 0.007), serum ferritin ≥ 1500 ng/ml (OR 12.3, 95% CI 3.1–49.6, *P* < 0.001), CRP ≥ 13 mg/L (OR 4.6, 95% CI 1.3–16.9, *P* = 0.021) and total GGO score ≥ 4 (OR 6.3, 95% CI 1.8–21.9, *P* = 0.003), were identified as independent predictors for RPILD. Cox regression model showed that total CT GGO score ≥ 4 (HR 4.8, 95% CI 1.3–17.9, *P* = 0.020), KL-6 > 1600 U/ml (HR 3.7, 95% CI 1.5–9.1, *P* = 0.004) and CRP > 5.8 mg/L (HR 3.7, 95% CI 1.0–12.8, *P* = 0.044) were poor prognostic risk factors, however initial combined treatment (HR 0.3, 95% CI 0.1–0.8, *P* = 0.019) predicted good prognosis in anti-MDA5 positive patients.

**Conclusion:**

Anti-MDA5 positive patients demonstrated a high prevalence of ILD on admission, leading to a high short-term mortality rate. Higher total GGO score, higher levels of initial KL-6 and CRP predict poor outcome in anti-MDA5 positive patients. However, initial intensive treatment may improve the prognosis.

## Background

In 2005, anti-melanoma differentiation-associated protein-5 (anti-MDA5) was identified as a novel autoantibody by Sato [1], in patients diagnosed with clinically amyopathic dermatomyositis (CADM), which was defined as having a manifestation of typical skin lesions of dermatomyositis (DM) without clear evidence of myopathy. Since then, a lot of studies have verified the association between anti-MDA5 and CADM [2, 3]. Accumulating evidences have demonstrated that patients with anti-MDA5 often have a high mortality rate caused by rapidly progressive interstitial lung disease (RPILD) [2–6]. While immunosuppressive treatment has improved the outcome of anti-MDA5 positive patients [7, 8], there still were patients failing to respond and dying of respiratory failure shortly after the diagnosis was established.

Risk factors predicting poor prognosis have been established in polymyositis (PM)/DM patients including age, serum ferritin level, skin ulcers, partial arterial pressure of oxygen (PaO_2_) and anti-MDA5 antibody [2, 9–11], among which anti-MDA5 antibody was consistently reported as poor prognostic risk factor in most studies [12]. Of note, in a study containing all types of myositis, anti-MDA5 has such a strong association with poor prognosis, that it dominates the analysis [11]. While in the subgroup of anti-MDA5 positive patients, the reported risk factors varied in different reports, possibly due to small sample size [5, 9, 13].

Therefore, we conducted a retrospective cohort of 90 anti-MDA5 positive patients, which involved in different departments including rheumatology, pneumonology and dermatology. This study was designed to summarize the clinic-biological characteristics and identify the prognostic factors in anti-MDA5 positive patients.

## Methods

### Patients

We conducted a retrospective study of patients who visited the First Affiliated Hospital of Zhengzhou University and were diagnosed with anti-MDA5 positive DM/CADM or PM for the first time, from September, 2018 to December, 2019. The exclusion criteria included: (1) age < 16 years old, (2) complicated with other connective tissue diseases, (3) complicated with lethal carcinoma that occurred before, 4) incomplete clinical or laboratory data necessary for this study. The diagnosis of classical DM and PM were based on Bohan and Peter criteria [14, 15], and CADM was based on Sontheimer criteria [16]. This study was conducted in accordance with the Declaration of Helsinki and approved by the Ethics Committee of the First Affiliated Hospital of Zhengzhou University (2020-KY-194).

### Data collection

A standard form was used to collect demographic, clinical and laboratory data from medical records. Data collection included the age of disease onset, gender, duration, and symptom. The laboratory data included the serum levels of creatine kinase (CK), lactate dehydrogenase (LDH), Krebs von den Lungen-6 (KL-6), ferritin, ESR, CRP and the titer of ANA. All patients were tested for a panel of myositis-specific antibodies (MSAs) and myositis-associated antibodies (MAAs) including anti-MDA5 and TIF1-γ antibodies using ELISA kits (MBL, Japan), and anti-OJ, EJ, PL7, PL12, SRP, Jo-1, Mi-2, Ku and Ro52 antibodies using lining immunofluorescence (Euroimmun, Germany) by following the manufacturer’s instructions.

The initial treatment regimens were also recorded according to medical records, including glucocorticoid (GC), calcineurin inhibitors (CNI) such as tacrolimus and cyclosporine A, cyclophosphamide (CYC), and intravenous immunoglobulin (IVIG). High-dose GC is defined of a prednisolone equivalent of 1 mg/kg daily. Initial combined treatment is defined as a combination of high dose GC and at least one immunosuppressant starting immediately after diagnosis. The main outcome was death. Survival status was respectively or prospectively confirmed by hospital records or the follow-up calls.

### HRCT findings

High-resolution computed tomography (HRCT) scan was performed in every patient on admission. After excluding infections, RPILD was defined as following: deteriorating dyspnea on exertion, decrease in PaO_2_ levels by > 10 mmHg within 4 weeks, or expanding GGO on HRCT within 4 weeks. HRCT findings were assessed both in GGO score and fibrosis score using the method proposed by Kazerooni et al. [17] Every patient’s HRCT was evaluated in three limited levels: the mid-aortic arch, tracheal bifurcation, and 1 cm above the diaphragm. Each lobe (right upper, middle, and lower, and left upper and lower lobes) was scored at the three sites on a scale of 0–5 as follows: GGO scores for GGO involving the lobe: 0, none; 1, ≤ 5%; 2, 5 to < 25%; 3, 25 to 49%; 4, 50 to 75%; and 5, > 75%. Similarly, fibrosis scores were assessed according to honeycombing involving the lobe: 0, none; 1, interlobular septal thickening without discrete honeycombing; 2, < 25%; 3, 25–49%; 4, 50–75%; and 5, > 75%. The images were independently reviewed by a pulmonary radiologist and a respiratory specialist (C. Y and W. SJ) blinded to the patients’ clinical information. Disagreement between two observers was solved by consensus. Total GGO scores and fibrosis scores were calculated by summing the scores of the five lobes.

### Statistical analysis

Hypothesis testing was performed for comparing continuous variables in different outcome groups using *t *test or Mann–Whitney U test. Categorical variables were assessed by the Chi-squared test or Fisher’s exact test.

All continuous variables were converted to dichotomous variables for univariate and multivariate analysis, and cut-off values were determined by receiver operating characteristics (ROC) [18]. Factors associated with RPILD were subjected to univariate logistic regression analysis. The predictive factors of RPILD with *P* < 0.1 in univariate analysis were included in a multiple logistic regression model. Variables were selected by a forward stepwise (likelihood ratio) procedure based on the *P*-value. Factors associated with poor outcome of anti-MDA5 positive patients were subjected to univariate analysis using the log rank test. Variables with *P* < 0.1 in the univariate analysis were sequentially included in the multivariate Cox regression analysis, and the forward stepwise (likelihood ratio) method was used to select the variables that were eventually included in the model. Kaplan–Meier analysis with the log-rank test was used for factors selected by final model for predictors for mortality. The concordance index (C-index) of Cox regression model was calculated using R package rms (ver 6.0-1).

Statistical analysis was performed using SPSS software (ver 20.0, USA), MedcCalc software (ver 18.2.1, Belgium) and R (ver 4.0.3). The significance levels were computed for 2-tailed testing and the cutoff of significance was set at *P* < 0.05.

## Results

### Baseline features and outcomes of anti-MDA5 positive patients

A total of 90 anti-MDA5 positive patients were included in our study. The demographic features, antibody variables and treatment regimens on admission, and the clinical outcomes were recorded in Table1. The average age at disease onset was 51.9 ± 12.1 years, and female accounted for 63.3% of the cohort. This cohort consisted of 70 (77.8%) CADM, 19 (21.1%) DM and 1 (1.1%) PM cases, among whom the CADM patients accounted for the majority. All the patients were negative for other MSAs except anti-MDA5 antibody. Meanwhile, 64 (71.1%) patients were also positive for anti-Ro52 antibody.

**Table 1:** Comparison of clinical features between survival group and death group

	Total patients (n = 90)	Survival group (n = 68)	Death group (n = 22)	*P* value
*Demographic features*				
Female, n (%)	57 (63.3)	43 (63.2)	14 (63.6)	0.973
Age, years, mean ± SD	51.9 ± 12.1	49.5 ± 11.4	59.2 ± 11.6	0.001*
Disease duration, months, median (IQR)	2.0 (1.0, 3.8)	2.0 (1.0, 4.0)	1.0 (1.0, 2.8)	0.086
*Clinical features*				
CADM, n (%)	70 (77.8)	51 (75.0)	19 (86.4)	0.380
Raynaud’s phenomenon, n (%)	9 (10.0)	7 (10.3)	2 (9.1)	1.000
Gottron’s papules, n (%)	61 (67.8)	46 (67.6)	15 (68.2)	1.000
Skin ulcer, n (%)	2 (2.2)	1 (1.5)	1 (4.5)	0.431
*Laboratory test*				
Serum ferritin, ng/ml, median (IQR)	875.0 (538.2, 1794.2)	734.5 (426.2, 1255.5)	2198.5 (1008.8, 2923.5)	< 0.001*
KL-6, U/ml, median (IQR)	939.5 (654.2, 1362.5)	857.0 (640.8, 1202.2)	1736.0 (830.0, 2958.5)	0.002*
CK, U/L, median (IQR)	64.5 (34.2, 122.5)	64.5 (34.0, 114.5)	72.5 (41.8, 132.2)	0.508
CRP, mg/L, median (IQR)	6.4 (2.8, 21.8)	4.4 (1.7, 16.5)	23.0 (11.2, 44.7)	< 0.001*
ESR, mm/h, median (IQR)	32.0 (22.0, 48.0)	30.5 (19.8, 45.0)	37.0 (27.2, 71.8)	0.043*
Lymphocyte, × 10^9^/L, median (IQR)	0.7 (0.6, 1.1)	0.8 (0.6, 1.2)	0.6 (0.4, 0.9)	0.014*
Anti-Ro52, n (%)	64 (71.1)	44 (64.7)	20 (90.9)	0.018*
Anti-MDA5, U/ml, median (IQR)	188.5 (158.5, 206.3)	187.5 (155.5, 206.0)	192.3 (165.8, 205.0)	0.333
ANA ≥ 1:320, n (%)	15 (16.7)	11 (16.2)	4 (18.2)	1.000
*HRCT findings*				
Total CT GGO score, median (IQR)	2.0 (0.0, 7.8)	0.5 (0.0, 4.0)	11.0 (5.0, 14.8)	< 0.001*
Total CT fibrosis score, median (IQR)	2.0 (1.0, 6.0)	2.0 (0.0, 4.0)	6.0 (2.2, 12.0)	0.001*
*Treatment*				
Initial combination therapy, n (%)	58 (64.4)	53 (77.9)	5 (22.7)	< 0.001*
IVIG, n (%)	43 (47.8)	31 (45.6)	12 (54.5)	0.465

CADM, clinically amyopathic dermatomyositis; KL-6, Krebs von den Lungen-6; LDH, lactate dehydrogenase; CK, creatine kinase; CRP, C-reactive protein; ESR, erythrocyte sedimentation rate; anti-MDA5, anti-melanoma differentiation-associated protein-5; ANA, anti-nuclear antibody; HRCT, high-resolution CT; GGO, ground-glass opacity; CNI, calcineurin inhibitors; CYC, cyclophosphamide; IVIG, intravenous immunoglobulin; SD, standard deviation; IQR, interquartile range

*Significant

Although almost all patients were diagnosed in early stage at a median time of 3.7 months, 81 (90%) patients already presented ILD on admission and 35 (38.9%) patients developed RPILD subsequently. During the first six months follow-up, a high mortality rate of 24.4% was observed in our cohort. Patients all died of respiratory failure caused by ILD, some of whom were complicated with infections in the end stage, at an average time of 6.6 ± 5.9 weeks (range 1–24 weeks) after diagnosis.

During the first six months follow-up, malignancies were found in three CADM patients: one with esophageal cancer, one with lung adenocarcinoma and one with thyroid carcinoma. However, no patient died of malignancy in the first six months.

### Comparison of clinical features between survivors and non-survivors

No significant difference was observed in other demographic features, except the non-survivors were significantly older at the age of disease onset than the survivors. As to the laboratory data, the death group presented a significant higher initial serum levels of ferritin, KL-6, LDH, ESR and CRP, suggesting a more intensive inflammation inside, than the survival group. The non-survivors had a significant higher incidence of anti-Ro52 positivity than the survivors, reminding physicians to pay attention to the patients who were double-positive for anti-MDA5 and anti-Ro52. Not surprisingly, the non-survivors had significant worse HRCT presentations on admission, both in total CT fibrosis score and total GGO score. Initial combination therapy was more common in the survivors, but the use of IVIG was of no significant difference between the two groups. (Table 1).

### Treatment regimens

All patients were treated with high-dose GC at diagnosis, of whom 58 cases were initially combined with CNI and/or CYC and 32 cases were step-up treated. In our cohort, CNI was a preferable choice than CYC but without significant difference (41.1% vs. 31.1%, *P* = 0.16). Only seven patients (7.8%) were treated with a triple combination of high-dose GC with CYC and CNI, due to the concern of serious infections. Additional IVIG (0.4 g/kg daily) was administered in 43 cases based on physician’s decision after comprehensive assessment of patients’ condition. (Table 2).

**Table 2:** Initial treatment regimens of 90 anti-MDA5 positive patients

Variables (n = 90)	Value
Initial combination therapy, n (%)	58 (64.4)
High dose GC + CNI, n (%)	30 (30.0)
High dose GC + CYC, n (%)	21 (23.3)
High dose GC + CNI + CYC, n (%)	7 (7.8)
Step-up therapy, n (%)	32 (35.6%)
*Additional treatment*	
IVIG, n (%)	43 (47.8)
Hemodialysis	2 (2.22)

anti-MDA5, anti-melanoma differentiation-associated protein-5; GC, glucocorticoid; CNI, calcineurin inhibitors; CYC, cyclophosphamide; IVIG, intravenous immunoglobulin

### Factors associated with RPILD in anti-MDA5 positive patients

In univariate analysis, factors including age, fever, serum ferritin, CRP, KL-6, lymphocyte, anti-MDA5, total GGO score and total fibrosis score were found significantly associated with RPILD. In logistic multivariate analysis, disease duration < 2 months (OR 6.1, 95% CI 1.7–22.4, *P* = 0.007), serum ferritin ≥ 1500 ng/ml (OR 12.3, 95% CI 3.1–49.6, *P* < 0.001), CRP ≥ 13 mg/L (OR 4.6, 95% CI 1.3–16.9, *P* = 0.021) and total GGO score ≥ 4 (OR 6.3, 95% CI 1.8–21.9, *P* = 0.003) were identified as independent risk factors for RPILD (Table 3).

**Table 3:** Initial parameters associated with RPILD using a logistic regression model

	Univariate analysis			Multivariate analysis		
	OR	95% CI	*P* value	OR	95% CI	*P* value
Age ≥ 55 years	5.5	2.2–14	< 0.001*	–	–	**–**
Disease duration < 2 months	4.3	1.7–10.7	0.002*	6.1	1.7–22.4	0.007*
Serum ferritin ≥ 1500 ng/ml	11.9	4–35.3	< 0.001*	12.3	3.1–49.6	< 0.001*
KL-6 ≥ 1600 U/ml	6.3	2–19.9	0.002*	–	–	–
CRP ≥ 13 mg/L	5.3	2.1–13.5	< 0.001*	4.6	1.3–16.9	0.021*
Lymphocyte < 0.5 × 10^9^/L	3.3	1.2–9.1	0.024*	–	–	–
Anti-MDA5 ≥ 190 U/ml	2.4	1–5.7	0.051	–	–	–
Total CT GGO score ≥ 4	10.2	3.8–37.5	< 0.001*	6.3	1.8–21.9	0.003*

KL-6, Krebs von den Lungen-6; CRP, C-reactive protein; anti-MDA5, anti-melanoma differentiation-associated protein-5; GGO, ground-glass opacity; * significant

### Factors associated with outcomes in anti-MDA5 positive patients

To identify the prognostic factors associated with death in anti-MDA5 positive patients, we performed univariate analysis using all the initial variables. In the univariate analysis, a number of variables, including demographic, clinical, laboratory and image parameters were found significantly related to death, while initial combination related to survival. In the multivariate Cox regression analysis, we identified total CT GGO score ≥ 4 (HR 4.8, 95% CI 1.3–17.9, *P* = 0.020), initial KL-6 > 1600 U/ml (HR 3.7, 95% CI 1.5–9.1, *P* = 0.004), CRP > 5.8 mg/L (HR 3.7, 95% CI 1.0–12.8, *P* = 0.044) as independent predictors for death, and initial combination (HR 0.3, 95% CI 0.1–0.8, *P* = 0.019)as a predictor for survival. (Table 4). This model proved a good model fitness (C-index = 0.88, 95% CI 0.81–0.95).

**Table 4:** Initial parameters associated with death using a Cox regression model

	Univariate analysis			Multivariate analysis		
	HR	95% CI	*P* value	HR	95% CI	*P* value
Age ≥ 55 years	5.0	2.1–11.9	< 0.001*	**–**	**–**	**–**
Disease duration < 2 months	2.1	0.9–5.2	0.088	**–**	**–**	**–**
Lymphocyte < 0.6 × 10^9^/L	2.5	1.0–6.4	0.047*	**–**	**–**	**–**
Serum ferritin ≥ 2000 ng/ml	14.9	5.2–42.6	< 0.001*	**–**	**–**	**–**
KL-6 ≥ 1600 U/ml	23.6	7.2–77.2	< 0.001*	3.7	1.5–9.1	0.004*
CRP ≥ 5.8 mg/L	5.1	2.2–11.9	< 0.001*	3.7	1.0–12.8	0.044*
ESR ≥ 23 mm/h	3.6	1.4–9.1	0.006*	**–**	**–**	**–**
Anti-Ro52	2.9	1.2–7.4	0.021*	**–**	**–**	**–**
Total CT GGO score ≥ 4	10.8	4.4–26.4	< 0.001*	4.8	1.3–17.9	0.020*
Total CT fibrosis score ≥ 10	49.7	11.4–216.1	< 0.001*	**–**	**–**	**–**
Initial combined treatment	0.1	0.04–0.3	< 0.001*	0.3	0.1–0.8	0.019*

KL-6, Krebs von den Lungen-6; CRP, C-reactive protein; ESR, erythrocyte sedimentation rate; GGO, ground-glass opacity

*Significant

**Fig. 1:**
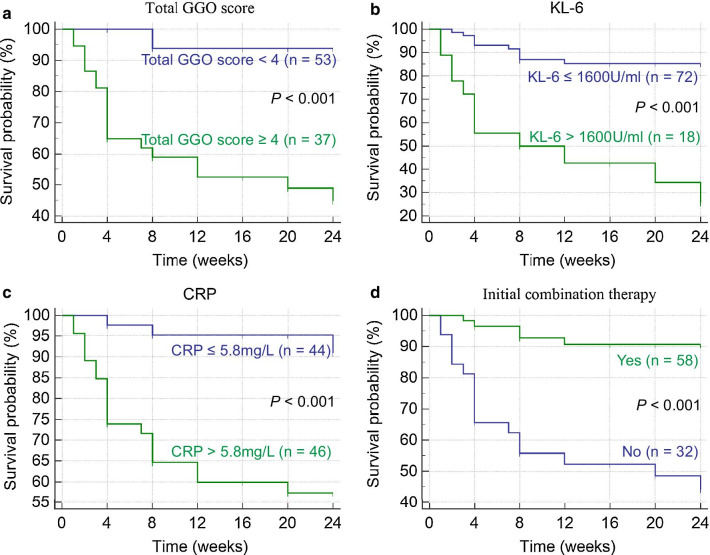
Survival curves of anti-MDA5 positive patients in each group based on initial KL-6, CRP level, total GGO score and initial combined treatment. KL-6, Krebs von den Lungen-6; CRP, C-reactive protein; GGO, ground-glass opacity

To better understand prognostic value of the aforementioned risk factors, the survival curves of patients divided by the aforementioned risk factors using Kaplan–Meier analysis were shown in Fig. 1.

## Discussion

It has been reported that there are two waves of fatal events in PM/DM patients, and the first wave of mortality is caused by ILD and infections in the early stage [19]. Our study has revealed a high prevalence of ILD in anti-MDA5 positive patients, consistent with previous studies in Europeans and Japanese [2, 20]. The short-term mortality rate of anti-MDA5 positive patients was especially high within the first 6–12 months [5, 9, 13]. Based on these findings, we set to analyze the first six months living situation and identify the predictive factors for death in anti-MDA5 positive patients. Factors including shorter disease duration, higher total GGO score, higher levels of serum ferritin and CRP were found independently associated with RPILD. The multivariate analysis has confirmed that higher total GGO score, higher levels of initial KL-6 and CRP are poor prognostic factors, and that initial combined treatment predicts favorable outcome in anti-MDA5 positive patients.

It has been widely accepted that MSAs, especially anti-MDA5 antibody, have an important role in predicting prognosis of PM/DM patients. When the cohort contains patients with all types of myositis, the significance of anti-MDA5 antibody in predicting death is dominating, leading to other potential risk factors ignored [11]. However, the predictive value may be reduced when MSAs were not completely examined in the cohort [21]. We would like to emphasize that every patient in our cohort was tested for a panel of important MSAs and MAAs, and that anti-MDA5 positive patients were recruited from different specialties to avoid a potential selection bias.

In the current study, both total GGO and total fibrosis score were significantly higher in the deceased group than the survival group. However, only total GGO score was identified as an independent predictor for RPILD and poor outcome in the multivariate analysis. This may be caused by the fact that honey combing, an important component of fibrosis scoring, is less common in the early stage of anti-MDA5 associated ILD. On the contrary, GGO was a quite common pattern in the early stage. Similarly, in a prospective study, patients with GGO in all six lung fields at baseline all died of respiratory failure during follow-up [5]. Fujiki et al. [22] also reported the prognosis of patients with right middle lung lobe GGO score ≥ 2 was poor. Different from the wide consensus on the utility of CT score, the effect of initial KL-6 level was still controversial in different reports [9, 11, 13, 21–23]. In our cohort, we found the cut off value of initial KL-6 > 1600 U/ml as an independent poor prognostic predictor and the cumulative six-months survival rate differed significantly. In addition, Ye et al. [13] reported initial KL-6 of 792 U/ml as the cut-off for discriminating between survivors and non-survivors in anti-MDA5 positive patients. Moreover, dynamic change of KL-6 level, like marked increase of serum KL-6 during the first four weeks, has also been reported as a poor prognostic predictor in PM/DM patients [23]. It could be explained by that higher total GGO score and KL-6 level both indicated a more rapid progression of ILD and wider extent of lung involvement, resulting in respiratory failure in the early stage.

The value of CRP in predicting death in anti-MDA5 positive patients was rarely reported before. CRP is a general reflection of inflammatory which has been widely tested clinically. In a large multicenter myositis-associated ILD cohort, CRP > 10 mg/L was revealed as an independent poor prognostic risk factor [11]. Moreover, a meta-analysis indicated that higher CRP level is associated with an increased risk of developing ILD in PM/DM patients [24]. Consistent with these findings, our study demonstrated higher initial CRP levels significantly associated with both RPILD and poor outcome in anti-MDA5 positive patients.

In this cohort, we found a high incidence of the positivity for antibodies against Ro52, which turned out to be a risk factor for poor prognosis in univariate analysis, in accordance to previous study [25]. In spite of their low specificity, anti-Ro52 antibodies are the most frequent autoantibodies of the MAA, being present in > 30% of IIM patients [26, 27]. Infantino [28] proposed when IIM was clinically suspected, patients with anti-Ro52 + /anti-Jo1- int the test for ENA should all test for MSA. Clinically, we did according to recommendation, which could explain the high incidence of positivity for anti-Ro52 in this cohort. However, the titer of anti-MDA5 antibodies at baseline wasn’t correlated with the prognosis. Currently, no literature is available regarding the direct effect of anti-MDA5 antibodies on organ damage. Further, Yoshiyuki and colleagues [29] did not detect significant difference regarding the initial decrease in anti-MDA5 titer in most cases, including the fatal case, suggesting that anti-MDA5 titer was not necessarily to be a useful marker for disease monitoring and prognosis predicting.

Different from previous studies, we didn’t find any association between CADM and poor prognosis in our study. This discrepancy was caused by the strong connection between CADM and anti-MDA5 antibody in the cohorts including all types of myositis, which apparently doesn’t exist in our cohort. Although initial serum ferritin wasn’t identified as an independent predictor for death, it was still significantly associated RPILD. In addition, shorter disease duration was also revealed as a predictor for developing RPILD in this study. Thirty-one patients had RPILD at their first hospitalization, and only four patients developed RPILD during follow-up, which suggests that the rapid progression of ILD mostly happens in the early stage of the disease. Different from a French anti-MDA5 positive cohort, no symptom was found associated with the prognosis [27].

More than half of the patients were treated with initial combination regimens, and it turned out the initial combination treatment significantly improve the prognosis, which was in accordance with a prospective study [7]. Owing to its nature of retrospective study, treatment regimens relied on attending physician’s decision, instead of a pre-determined protocol. Some of the patients with diffuse GGO on HRCT, which was difficult to be distinguished from infections, were treated with step-up regimens due to the concern of severe infections. This may lead to exaggeration of the advantages of initial combined treatment. Initial treatment regimens were not included into the risk factor analysis for RPILD owing to the majority of RPILD cases occurred before therapy were initiated. Although IVIG was reported effective in treating PM/DM associated ILD [30], such efficacy was not observed in our study.

There are several limitations of this study. First, patients were all recruited from a single center of a tertiary hospital which may lead to a bias of a more severe form of this disease. Second, pulmonary function test, which has been reported as a potential prognostic factor, was not included into analysis, since many patients suffered a severe chest congestion and were unable to complete the lung function tests.

## Conclusions

In conclusion, we observed that anti-MDA5 positive patients presented a high prevalence of ILD on admission, resulting in a high short-term mortality rate. Higher total GGO score, higher levels of initial KL-6 and CRP were identified as poor prognostic factors in anti-MDA5 positive patients, however initial intensive treatment may improve the prognosis.

## Data Availability

Data can be requested from the corresponding author.

## Abbreviations

ANA: Anti-nuclear antibody
anti-MDA5: Anti-melanoma differentiation-associated protein-5
CADM: Clinically amyopathic dermatomyositis
CK: Creatine kinase
CNI: Calcineurin inhibitors
CRP: C-reactive protein
CYC: Cyclophosphamide
DM: Dermatomyositis
ESR: Erythrocyte sedimentation rate
GGO: Ground-glass opacity
HR: Hazard ratio
HRCT: High-resolution CT
ILD: Interstitial lung disease
IQR: Interquartile range
IVIG: Intravenous immunoglobulin
KL-6: Krebs von den Lungen-6
LDH: Lactate dehydrogenase
PaO_2_: Peripheral capillary oxygen saturation
PM: Polymyositis
RPILD: Rapidly progressive interstitial lung disease
SD: Standard deviation
